# Combining Cognitive and Sensorimotor Assessments to Facilitate Detection of Age-Related Cognitive Decline

**DOI:** 10.1109/TNSRE.2026.3711558

**Published:** 2026

**Authors:** Wei Yin, Giovanni Oppizzi, Raziyeh Baghi, Meizhen Huang, Kyung Koh, Li-Qun Zhang

**Affiliations:** Department of Physical Therapy and Rehabilitation Science, University of Maryland, Baltimore, MD 21201 USA. He is now with the School of Applied Engineering and Technology, New Jersey Institute of Technology, Newark, NJ 07102 USA; Department of Bioengineering, University of Maryland, College Park, MD 20742 USA; Department of Physical Therapy and Rehabilitation Science, University of Maryland, Baltimore, MD 21201 USA; Department of Physical Therapy and Rehabilitation Science, University of Maryland, Baltimore, MD 21201 USA. She is now with the Department of Rehabilitation Sciences, The Hong Kong Polytechnic University, Hong Kong; Department of Physical Therapy and Rehabilitation Science, University of Maryland, Baltimore, MD 21201 USA. He is now with the Department of Bioengineering, University of Maryland, College Park, MD 20740 USA; Department of Physical Therapy and Rehabilitation Science and the Department of Orthopaedics, University of Maryland, Baltimore, MD 21201 USA, and also with the Department of Bioengineering, University of Maryland, College Park, MD 20740 USA

**Keywords:** Cognitive status, combined cognitive and sensorimotor evaluation, eye tracking, anti-saccade, eye movements

## Abstract

Early detection of cognitive decline is critical for reducing progression of neurodegenerative diseases. Bioimaging- and biofluids- based approaches are invasive and expensive; clinical screening tools have difficulties in discerning subtle cognitive changes. Eye tracking measures have been used to detect cognitive deficits effectively and reduction in sensorimotor performance may precede cognitive declines by several years. Therefore, we developed a combined cognitive and sensorimotor evaluation system by integrating an arm-hand exoskeleton and an eye-tracking device to facilitate the early detection of cognitive decline. Three combined cognitive and sensorimotor tests were investigated: combined anti-saccade motor test (CASMT), combined anti-saccade sensory test (CASST), and combined trail-making test B (CTMT-B). The combined tests were compared to conventional anti-saccade test (AST). Thirty-two healthy subjects, including 16 young subjects and 16 older adults, participated in the study. Results showed that the combined assessment better detected cognitive differences between older and young subjects than cognitive (eye-tracking) test alone. Specifically, 1) in CASMT, older subjects exhibited significantly longer eye movement latency than the young subjects (p < 0.001), while AST did not show such a difference. 2) in CASST, older adults showed longer latency in both eye (p = 0.003) and wrist (p = 0.005) movement than young subjects. 3) in CTMT-B, older adults took longer time to reach the targets (p < 0.001) and dwell longer at the targets (p < 0.001) than young subjects; older adults also showed more saccades during hand cursor reaching and dwelling, and lower cursor movement smoothness (p < 0.005). Furthermore, classification algorithms were used to predict participants’ age groups, with the top six best-performing models confirming that sensorimotor outcome measures from CTMT-B, particularly the cursor movement smoothness, are highly influential, indicating the combined approach improves detection of cognitive changes. Correlation analysis further confirmed statistically significant negative correlation coefficients between MoCA results and outcome measures from CASMT and CTMT-B. The combined cognitive and sensorimotor tests showed more sensitive detections of cognitive and sensorimotor changes than the tests based on eye-tracking alone, and can potentially facilitate early detection of cognitive declines.

## I. Introduction

Alzheimer’S Disease and related dementias (ADRD) impact an estimated 6.7 million adults in the United States, with projections indicating a rise to 13.8 million by 2060 [1]. ADRD represents a progressive neurodegenerative condition currently devoid of a cure [1]. Optimal mitigation of ADRD progression and the preservation of cognitive function are most achievable when interventions are administered early in the disease process [1], [2].

Imaging- and biofluids-based biomarkers are validated to identify ADRD, which are inappropriate for general medical practice because of their invasive, high cost, and limited availability [3]. Instead, neuropsychological evaluations are the most popularly adapted approach used in clinics to screen cognitive impairment and provide an index of cognitive function [4], [5]. These evaluations range from brief screening tests to detailed neuropsychological batteries. Brief cognitive screening tests, including the Mini-Mental State Examination (MMSE), Mini Cognitive Assessment Instrument (Mini-Cog), and Montreal Cognitive Assessment (MoCA), have been widely utilized and refined in clinical practice in recent years as they can enable fast, high-patient-volume screening with minimal training [4]; however, they are facing challenges in sensitively characterizing various forms of mild cognitive impairment (MCI) and ADRD due to their inability to discern subtle cognitive differences [1], [6]. The neuropsychological test batteries are believed to be the gold standard for cognitive examination, which requires in-depth training to ensure accurate interpretation of the results [5]. Therefore, an easy-to-use, non-invasive, and sensitive screening tool for detection of MCI/ADRD is necessitated to promote the early identification and treatment of patients.

Cognitive and sensorimotor alterations are intricately linked, given the interdependent nature of human sensory input, motor function, and cognitive modulation in interacting with the environment [7], [8]; when one neural pathway is impaired, the function of the other neural systems will be altered as well [8]. Studies have also reported that sensorimotor changes may precede the onset of cognitive symptoms of AD by several years [9], [10], [11]. Consequently, human sensorimotor changes hold means as potential biomarkers for early detection of MCI/ADRD progression [8]. Furthermore, although recent research has demonstrated the capacity of saccadic eye movements to detect subtle cognitive declines [12], [13], [14], the integration of multiple assessment modalities through multimodal and multivariate approaches may yield more comprehensive insights in the diagnosis of MCI/ADRD, enhancing MCI/ADRD identification and classification [15], [16], [17]. Combining cognitive and sensorimotor modalities enables detection of executive and integrative deficits that single cognitive tasks may not fully capture. Although tests such as the anti-saccade task assess inhibitory control and attentional allocation, they do not evaluate how individuals coordinate cognitive operations with motor planning and execution through shared cortical-subcortical networks. By requiring simultaneous task performance, this approach can reveal deficits in divided attention, visuomotor integration, and executive resource allocation that may remain undetected in single-modality designs. In fact, difficulties in orchestrating simultaneous tasks have been confirmed to be associated with cognitive impairments in older adults [18], [19], [20], with high sensitivity and specificity in identifying cognitive status; nevertheless, the motor tasks used, gait [18], [20], [21] and upper extremity function test [19], are physically demanding and may lead to high fall risks for older adults, and the cognitive tasks, e.g., subtracting serial 7s [18] and counting back by one [19], are parts of the standardized tests in the neuropsychological test batteries that can provide limited information of participants’ cognitive status. Thus, there remains a lack of integrated, high-precision assessment frameworks capable of quantifying dual-task interference, visuomotor integration, and motor execution quality with strong sensitivity to capture subtle declines.

Therefore, this study developed a multimodal quantitative assessment technique by integrating an eye-tracking device and an arm-hand wearable robot. The wearable robot enables precise quantification of sensorimotor responses during dual-tasking through torque sensing and joint-motion tracking, as detailed in the Methods section [22]. Three noninvasive and practical combined cognitive and sensorimotor tests were developed based on two cognitive assessment tests, namely the trail-making test B (TMT-B) and the anti-saccade test (AST). The proposed dual-tasks were designed to combine eye and wrist responses under cognitive demand, requiring participants to inhibit reflexive responses, coordinate visuomotor actions, and manage simultaneous cognitive and motor tasks. By integrating AST- and TMT-B-based cognitive challenges with arm motor responses, this design may reveal deficits in inhibitory control, visuomotor integration, and dual-task coordination that may not be captured by single-modality assessments alone [13], [18], [19], [20], [21], [23], [24], [25]. Our design thus creates a controlled and quantifiable way to investigate executive resource allocation and sensorimotor integration under cognitive stress, which are among the earliest neural processes disrupted in MCI/ADRD.

Demonstrating that the proposed approach can effectively detect cognitive declines is a necessary foundational step before applying this framework to individuals with MCI/ADRD. Aging is associated with well-characterized declines in inhibitory control, attentional allocation, sensorimotor integration, and motor planning. Therefore, to initially assess the advantage of the proposed approach in discerning cognitive status difference, sixteen healthy older and sixteen healthy young participants were recruited. By characterizing these age-associated differences, the present work could establish an elemental methodological foundation for applying this system to individuals with MCI/ADRD. We hypothesized that the proposed combined cognitive and sensorimotor tests could better detect the cognitive differences between healthy older and young subjects than the cognitive (eye-tracking) test alone. A preliminary version of this work has been reported at the 2024 Biomedical Engineering Society Annual Meeting [26].

## II. Materials and Methods

### A. The Combined Cognitive and Sensorimotor Evaluation System

**Fig. 1.**
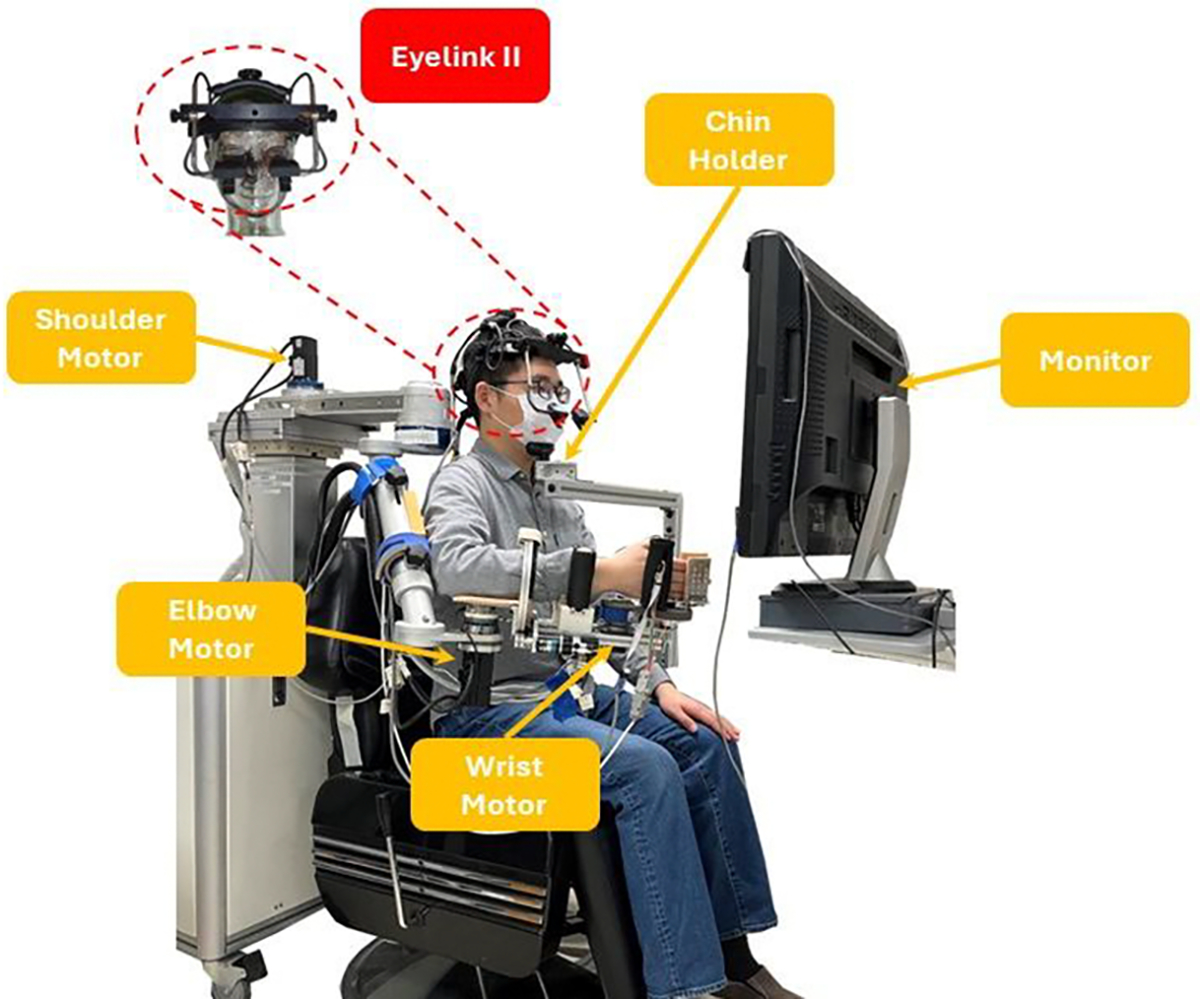
Experimental setup.

A novel system is developed by integrating a multi-joint upper limb exoskeleton [22] and a head-mounted eye-tracking camera (Eyelink II, SR Research Ltd., Kanata, ON, Canada) (Fig. 1). The high-speed eye-tracking camera can capture accurate saccadic data. The robot independently controls and measures shoulder, elbow, and wrist motions and records joint torques through integrated force sensors (Fig. 1). Two control modes were used in this study: an active mode, in which the robot maintained or imposed predefined joint motion, and a back-drivable mode, in which participants moved the robot with minimal resistance while joint kinematics were recorded [27], [28]. The robot and eye-tracking systems were synchronized for time-aligned measurement. Additional technical details of the robot have been reported previously [22].

### B. The Combined Cognitive and Sensorimotor Tests

Three combined cognitive and sensorimotor tests are created, including the Combined Anti-saccade Motor test (CASMT), Combined Anti-saccade Sensory test (CASST), and Combined Trail Making Test B (CTMT-B).

**Fig. 2.**
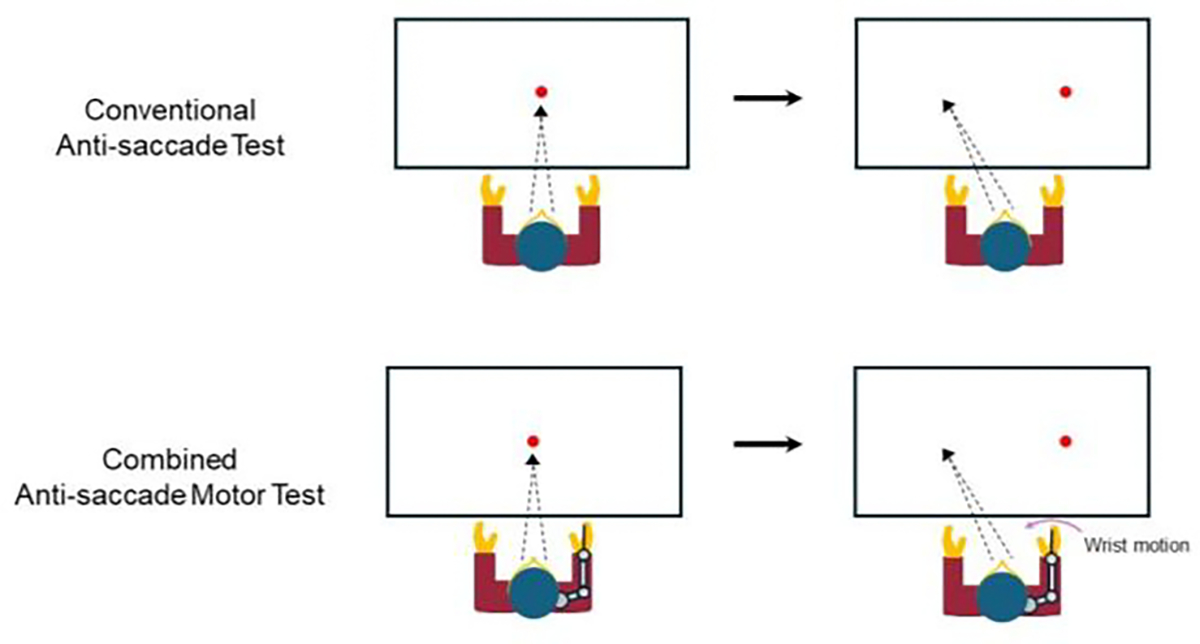
The Anti-saccade test (AST, upper row) and the Combined Anti-saccade Motor test (CASMT, bottom row).

CASMT: the CASMT is based on the popularly used Anti-saccade test (AST) in which subjects are asked to fixate on a motionless target and make a saccade in the direction away from the stimulus that is presented to one side of the target (Fig. 2, upper row). However, in CASMT, the subjects perform AST with the right arm wearing the robot, and flex/extend their wrist joints to the direction they look to simultaneously (Fig. 2, bottom row). During this test, the robot is in active mode with zero velocity so that the robot is fixed at a predefined posture that is 20° shoulder horizontal adduction, 60° elbow flexion, and 0° wrist flexion. Because the robot fixed the wrist position, participants could not physically flex or extend the wrist. Their attempted movements instead generated isometric torque, which was measured to quantify motor reaction time.

**Fig. 3.**
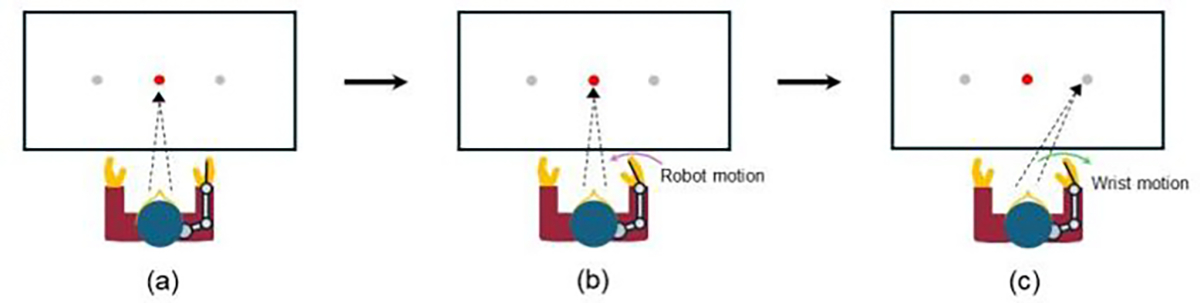
The Combined Anti-saccade Sensory test (CASST).

CASST: subjects are asked to fixate on a target in the center of the screen with a wearable robot on the right wrist (Fig. 3a). The robot flex or extend the wrist joint slowly (Fig. 3b). The subjects are asked that as soon as they feel the wrist joint motion, they look to the point in the opposite direction to the robot’s motion and flex or extend the wrist joint in the direction opposite to the felt motion to resist the robot’s movement simultaneously (Fig. 3c). Similar to CASMT, the subjects cannot actually resist the robot’s motion. But we could use the joint torque data to quantify subjects’ sensory reaction time.

**Fig. 4.**
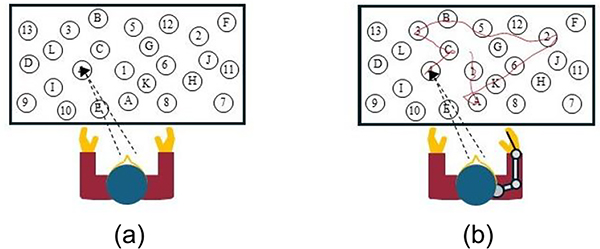
(a) The Trail Making Test B (TMT-B) and (b) the proposed Combined Trail Making Test B (CTMT-B). The red curve represents the cursor trajectory.

CTMT-B: the proposed CTMT-B is developed based on the TMT-B in which subjects look at a series of numbers (1 to 13) and letters (A to L) in ascending order and alternate between numbers and letters (Fig. 4a). But in CTMT-B, the subjects are asked to conduct TMT-B while manipulating the cursor on the screen to the numbers or letters they focus on simultaneously (Fig. 4b) through moving the robot which is in back-drivable mode. The hand cursor movement trajectory during the test remains on the screen during the trial (red lines in Fig. 4b).

### C. Subjects

Sixteen right-handed healthy young (eight men and eight women, age: 28 ± 3.9) and sixteen right-handed healthy older (seven men and nine women, age: 65 ± 6.6) subjects who had normal visual acuity (20/20 or better), either unaided or achieved through professional correction, without any history of upper extremity musculoskeletal disorders were recruited. The study protocol complies with the 1964 Helsinki Declaration and later amendments and was approved by the Institutional Review Board of the University of Maryland, Baltimore (HP-00089498). Upon obtaining written informed consent, a researcher conducted MoCA for the subjects [29]. All the recruited older subjects had a score of higher than 26 (27.3 ± 1.4), suggesting that they have normal cognitive functions. The MoCA of young subjects were 28.6 ± 1.3. Because the normality assumption was violated, the Mann-Whitney U test was used to compare MoCA scores between the two age groups, confirming that the young adults exhibited significantly higher MoCA scores than the older adults (*p* = 0.002). The subjects were given sufficient time to be familiar with the test protocols.

### D. Experimental Protocol and Data Collection

As shown in Fig. 1, the subject sat upright comfortably on a sturdy barber’s chair and wore the robot with joint alignment following [22]. The monitor was placed 60 cm in front of the subjects with vertical height adjusted to match individual eye level, and a chin holder was used to keep the head steady to ensure consistency of the eye-tracking results. The participants then completed AST, CASMT, CASST, and CTMT-B with enough time for resting between tests being offered. The eye tracker was calibrated and validated before each task using the standard EyeLink II 9-point procedure and recalibrated when needed. The robot joint angles and reaction toques were monitored in real-time with a sampling frequency of 1000 Hz; the eye tracking camera captured the saccadic data with sampling at 500 Hz. The details of each test are listed below.

AST and CASMT: the central fixation target was displayed in white on a black background for one second. After a 200ms blank interval, the saccade distractor appeared in a random order 7 cm or 14 cm away from the fixation target on either the left or right side for two seconds. Twenty trials were conducted for AST and CASMT.

CASST: three fixed targets were displayed in white on a black background with one in the center and two at 7 cm to the center on the left and right side. The robot randomly flexed/extended the subjects’ wrist joints at a slow speed of 0.5°/s, starting at an unknown time to the subjects. The velocity is determined based on our previous studies, in which we identified the appropriate velocity for proprioception tests [30], [31], [32], [33], [34]. Eight trials were conducted for this test.

CTMT-B: the cursor was initiated at the central screen when the test started. Two trials were conducted for this test during which a researcher would record the incorrect eye or cursor movement.

During all the tasks, trials were not paused when an error occurred, and participants were not instructed to correct the error. Verbal cueing was only provided during the practice trials for each task to ensure that participants fully understood the procedures. For CTMT-B, brief corrective feedback was also given during the first trial, which was not included in the analysis. No verbal instructions, feedback, or interactions occurred during any trial used for performance evaluation, ensuring that the recorded cognitive and motor metrics were not influenced by external attentional interruptions.

### E. Data Analysis

#### 1) Outcome Measures:

The saccadic data and the robot’s wrist joint torque profiles were used to derive outcome measures on subjects’ cognitive and sensorimotor performance. Details of the detection of saccade onsets and wrist motion onsets are described in the Supplementary Material and Fig. S1.

For AST and CASMT, the eye movement latency (EyeAST and EyeCASMT) of the correct response defined by the time from the target onset (see Figs. 2&S1) to the onset of the saccade was used to evaluate cognitive performance. Similarly, for CASMT, the wrist motion latency of the correct response (WristCASMT) was measured using the time from the target onset to the onset of the wrist motion and employed to quantify the motor performance. Only the saccades and wrist movements made within the time window 80–700 ms after target onset were included to avoid ‘anticipatory saccade/movement’ [13]. No trials from young participants were removed based on this criterion, and only two CASMT trials from one older participant, representing less than 1% of all trials, were excluded. Thus, no systematic group differences in trial removal were observed. The first four trials were also excluded to minimize learning effects. For CASST, the first two trials were excluded from data analysis to ensure reliability. The eye and wrist movement latencies (EyeCASST and WristCASST) of correct responses were calculated and they were defined by the time from the onset of robot motion (see Fig. 3b) to the onset of saccades and wrist motion (see Figs. 3c&S1), respectively. The onset of robot motion was determined by the wrist joint angular velocity reaching 0.5°/s, the predefined velocity for proprioception tests.

For CTMT-B, only the second trial was employed to evaluate subjects’ performance. The first trial was treated as a familiarization trial, with brief corrective feedback provided as needed to ensure task understanding. Excluding this trial minimized familiarization effects and allowed the second trial to better reflect stable CTMT-B performance. The hand-cursor movement trajectory (see Fig. 4b) was used to compute the mean reach time (average time the cursor moved between targets, tReach) and mean dwell time (average time the cursor was located on targets, tDwell) to evaluate the overall performance. Meanwhile, the average number of saccades when the hand-cursor dwelled on targets (nDwell) and when the hand cursor moved between targets (nReach) were computed for cognitive performance assessment. Details of the determination of cursor dwell and reach are described in the Supplementary Material and Fig. S2. Furthermore, the hand-cursor movement smoothness (average number of cursor motion speed peaks during reaching movements, nPeaks) was used to assess subjects’ motor function.

Short gaps in gaze data were reconstructed using linear interpolation, the default EyeLink II method and a common approach for handling brief, isolated signal loss in eye-tracking research. Trials with more than 20% missing data were excluded; only one older adult exceeded this threshold, while all other participants had less than 5% missing data. The initial trials of each task were excluded from data analysis to minimize the learning effect, which refers to the phenomenon where participants improve their performance during early trials as they become more familiar with the task. The number of initial trials excluded for each task was empirically determined through pilot testing, as no established guidelines define learning stabilization for the tasks used in this study. Because the tasks differed in total trial counts, exclusions were selected to remove a comparable proportion of early trials across tasks, ensuring that analyzed data reflected stable task performance rather than learning effects, thereby improving the reliability and generalizability of the findings [35], [36].

#### 2) Statistical Analysis:

A multivariate analysis of variance (MANOVA) was used to identify if significant performance differences exist between two age groups, with the multivariate normality being checked by the Mardia’s test [37]. A significance level of 0.05 was used for MANOVA; if significant differences were identified, *post-hoc* comparison of each cognitive and sensorimotor performance between young and older participants would be performed using the two-sample t-test with the Bonferroni correction being employed to reduce the likelihood of false positives. A paired t-test was utilized to compare the cognitive performance between AST and CASMT of participants within the same age group; the normality assumption was assessed using the Shapiro-Wilk test [38].

The outcome measures were divided into two categories, namely Eye Movement Measures (EM) and Sensorimotor Measures (SM). EM includes EyeAST, EyeCASMT, EyeCASST, nDwell, and nReach; SM consists of WristCASMT, WristCASST, tReach, tDwell, and nPeaks. Four machine-learning algorithms were used to build classification models to predict participants’ age groups, including Ridge Logistic Regression (RLR), Random Forest (RF), Support Vector Machine (SVM) with a linear kernel, and Extreme Gradient Boosting (XGB). Constructing and comparing classification models could provide a proof-of-concept demonstration of the discriminative value of the extracted multimodal metrics and further allow examination of feature importance, highlighting which metrics or which type of metrics contribute more strongly to detecting cognitive differences. The reason for choosing those models is that they are simple models with fewer parameters or more lightweight structures than neural network-based or deep learning models, leading to reduced risk of overfitting; the use of RLR is because the multicollinearity of predictors is detected. A nested leave-five-subject-out cross-validation was utilized to compute and compare the performance of each model using each measurement group and all measurements; to avoid data leakage, the data standardization was embedded within the nested cross-validation framework. For each outer leave-five-subject-out split, the mean and standard deviation of each feature were calculated using only the training data, and these parameters were then applied to standardize both the outer training set and the held-out test subjects. Within the outer training set, hyperparameter tuning was performed using an inner leave-five-subject-out cross-validation procedure, in which standardization parameters were again estimated only from each inner training fold and applied to the corresponding inner validation fold. The rationale of utilizing this approach is that the hyperparameter tuning and model evaluation are conducted with separate sets of data, reducing the risk of overfitting to individual subjects and enabling a more reliable estimate of the model’s generalization performance. The average F1 score and classification accuracy obtained through the nested leave-five-subject-out cross-validation approach were used as the model performance metrics. All statistical analysis was conducted in R and the hyperparameter tuning was performed using the caret package [39].

## III. Results

### A. Results of MANOVA

Two subjects’ data were removed in this test because one older subject did not complete the CTMT-B test caused by the conflict of the subject’s schedule and one older subject’s gaze data were missed during more than 20% of the CTMT-B test.

Multicollinearity of the performance metrics was detected by the correlation matrix, as the correlation coefficient between EyeCASST and WristCASST was higher than 0.9. Therefore, we excluded the data of EyeCASST from the test. The Mardia’s test confirmed that multivariate normality assumption was not violated as either the multivariate skewness (*p* = 0.36) or kurtosis (*p* = 0.72) results were not significant.

MANOVA test identified significant performance differences (Pillai’s trace equals to 0.78, *p* < 0.001); excluding WristCASST also led to a *p*-value of lower than 0.001 (Pillai’s trace equals to 0.81). Therefore, we then performed *post-hoc* comparison of each cognitive and sensorimotor performance between young and older participants and a significance level of 0.005 was used according to the Bonferroni correction.

### B. AST and CASMT

**Fig. 5.**
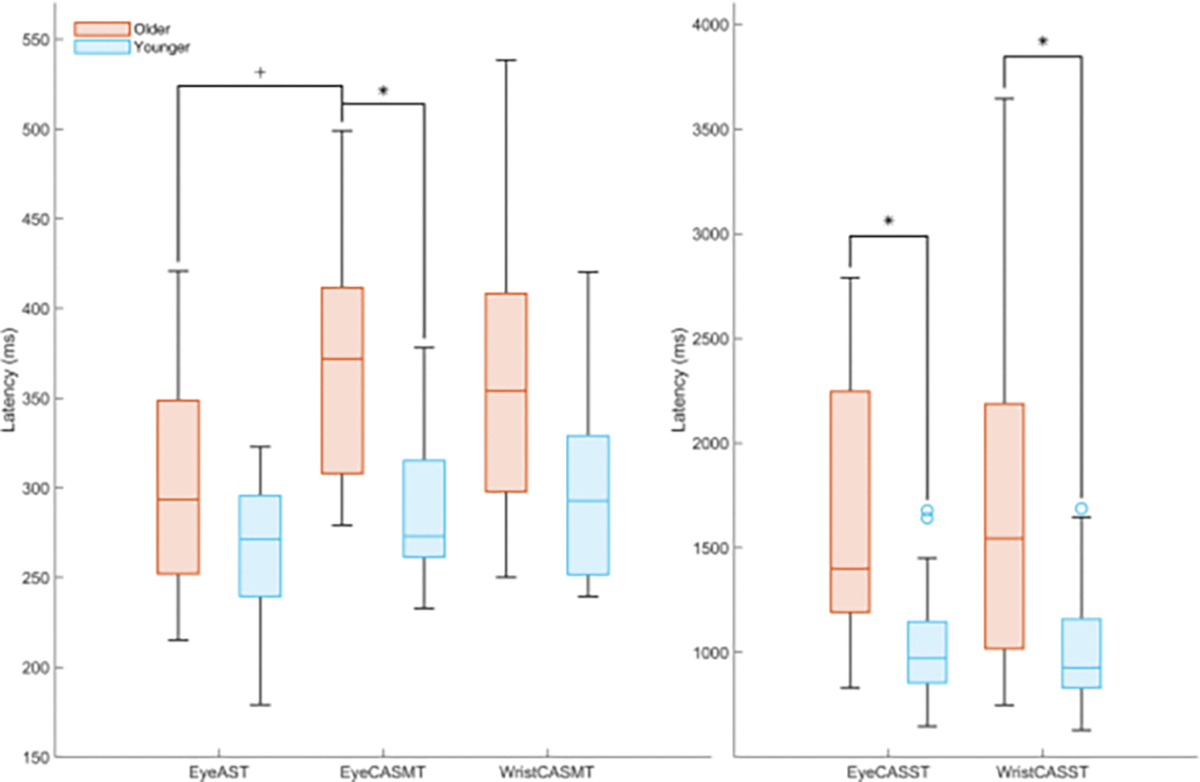
The outcome measures during AST, CASMT, and CASST of the older and young participants. * means p < 0.005; + means p < 0.05.

CASMT showed better performance than AST in identifying differences in the eye and wrist movement latency between older and young participants. The two-sample t-test showed that older subjects exhibited significantly longer latency in eye (*p* < 0.001, t_30_ = 4.77, mean difference: 90.2ms, 95% CI [51.6ms, 128.8ms]) movements during CASMT, whereas no significant difference between older and young subjects was identified in the eye movement latency (*p* = 0.056, t_30_ = 1.99, mean difference: 37.1ms, 95% CI [−0.9ms, 75.1ms], Cohen’s d = 0.68) during AST (Fig. 5). No significant differences in wrist movement latency were detected (*p* = 0.014, t_30_ = 2.62, mean difference: 68.3ms, 95% CI [15.1ms, 121.5ms]).

For the paired t-test, no violation to the normality assumption of the data from both older (*p* = 0.63) and young (*p* = 0.50) subjects was found by the Shapiro-Wilk test. The paired t-test confirmed that older subjects showed significantly increased eye movement latency during CASMT compared with AST (*p* = 0.001, t_15_ = 3.90, mean difference: 72.1ms, 95% CI [32.6ms, 111.5ms]), whereas no such significant changes were observed (*p* = 0.245, t_15_ = 1.21, mean difference: 19.0ms, 95% CI [−14.5ms 52.5ms]) in young subjects (Fig. 5). This 72.1ms increase can be denoted as the Dual-Task Cost (i.e., the additional processing time required when cognitive and sensorimotor demands must be coordinated simultaneously), which is consistent with our theoretical premise that dual-tasks introduce greater competition for shared executive and sensorimotor resources, leading to delayed response initiation.

### C. CASST

Older subjects exhibited significantly longer eye (*p* = 0.003, t_30_ = 3.22, mean difference: 757.4ms, 95% CI [277.7ms, 1237.2ms]) and wrist (*p* = 0.005, t_30_ = 3.08, mean difference: 645.7ms, 95% CI [216.9ms, 1074.5ms]) movement latency than young subjects (Fig. 5).

### D. CTMT-B

One older subject did not complete this test because of the conflict of the subject’s schedule. Therefore, data from ten young and ten older subjects were captured and analyzed. No errors were observed during the second trial of all twenty subjects. An example of the measurement of CTMT-B is shown in Fig. S3.

**Fig. 6.**
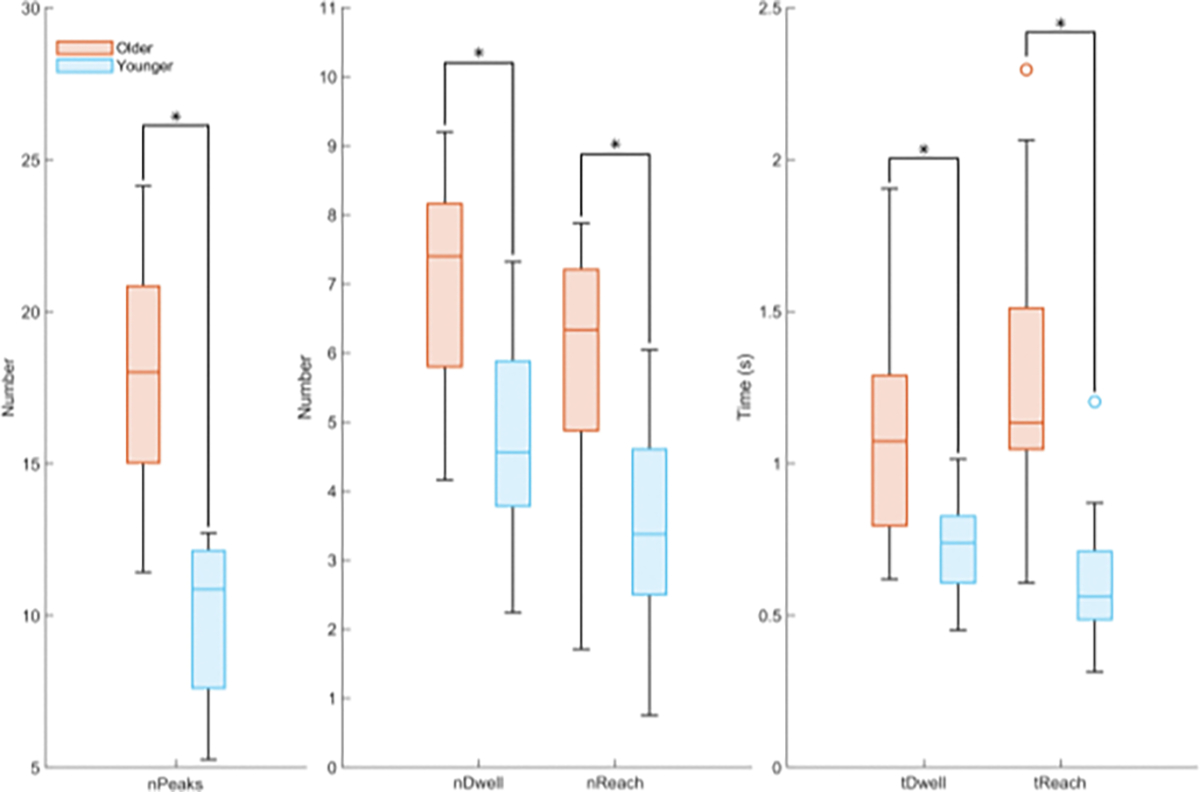
The outcome measures during CTMT-B. * means p < 0.005.

As shown in Fig. 6, the older subjects exhibited significantly increased tReach (*p* < 0.001, t_29_ = 5.18, mean difference: 0.656s, 95% CI [0.397s, 0.915s]) and longer tDwell (*p* < 0.001, t_29_ = 3.94, mean difference: 0.371s, 95% CI [0.179s, 0.564s]) during the test, compared to the young subjects.

On the cursor movement smoothness measurement, the older subjects showed a significantly more velocity peaks (nPeaks) than young subjects (*p* < 0.001, *t*_29_ = 7.02, mean difference: 8.02, 95% CI [5.67, 10.4]) (Fig. 6). Regarding the number of saccades, one subject’s data were excluded because gaze data were missed during more than 20% of the test; the older subjects adults showed significantly more nDwell (*p* < 0.001, *t*_28_ = 4.16, mean difference: 2.34, 95% CI [1.19, 3.49]) and nReach (*p* < 0.001, *t*_28_ = 3.86, mean difference: 2.83, 95% CI [1.33, 4.34]) than the young subjects (Fig. 6).

### E. Correlations Between the Outcome Measures

**Fig. 7.**
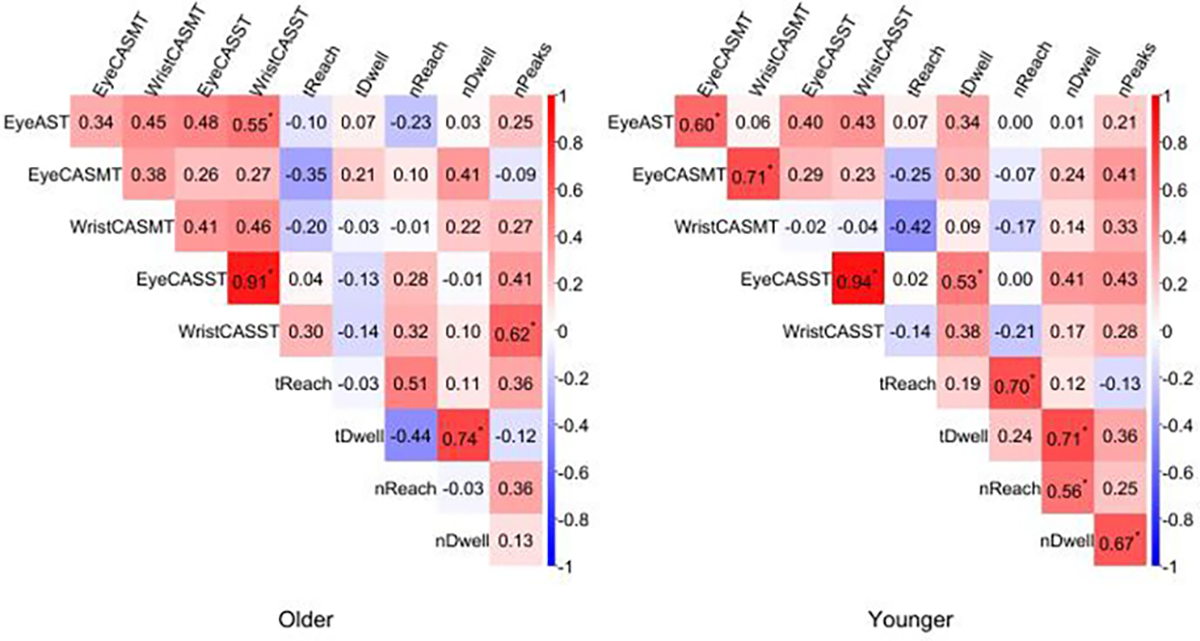
Pearson’s correlation coefficients between each pair of the outcome measures from older (left) and young (right) participants. * means p < 0.05.

The Shapiro-Wilk test found no normality violations (*p* > 0.05); thus, Pearson’s correlation coefficients between each pair of the outcome measures were calculated and summarized in Fig. 7. Significant correlations between EyeCASST and WristCASST (*r* = 0.91 and *r* = 0.94) and between tDwell and nDwell (*r* = 0.74 and *r* = 0.71) were identified for both older and young subjects. Furthermore, the young subjects exhibited significant correlations between EyeAST and EyeCASMT (*r* = 0.68), EyeCASMT and WristCASMT (*r* = 0.71), EyeCASST and tDwell (*r* = 0.53), tReach and nReach (*r* = 0.70), nReach and nDwell (*r* = 0.56), and nDwell and nPeaks (*r* = 0.67); the older subjects showed significant correlations between WristCASST and nPeaks (*r* = 0.62), and EyeAST and WristCASST (*r* = 0.55).

Pearson’s correlation coefficients between the proposed measures and MoCA scores are summarized in Table I. All the measures exhibited negative correlations with the MoCA scores, with EyeCASMT (*r* = −0.47, *p* = 0.006), tDwell (*r* = −0.45, *p* = 0.012), nDwell (*r* = −0.46, *p* = 0.011), and nPeaks (*r* = −0.43, *p* = 0.017) being statistically significant.

### F. Classification Models

The performance of each machine-learning model with each outcome measure set is listed in Table II. Results suggested that SM outperformed EM, no matter which classifier was used to construct the model. The XGB model using SM achieved the best model performance (accuracy: 0.87, F1: 0.87), followed by the RF model using SM (accuracy = 0.87, F1 = 0.83).

**Fig. 8.**
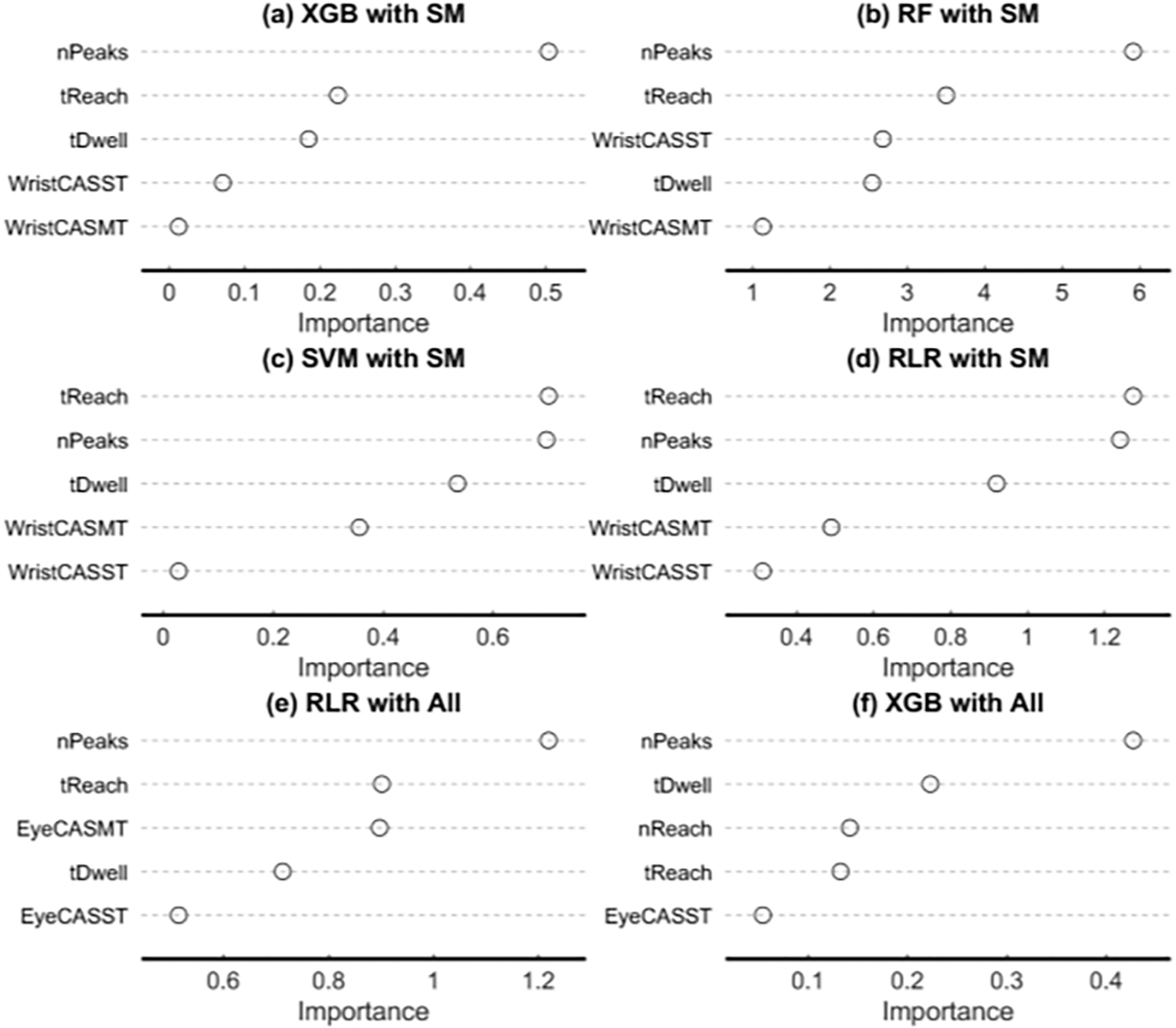
Feature importance of the top six best-performing models. Only the top five influential features of the XGB and RLR models with all measures are shown.

We then analyzed the feature importance of the top six best-performing models that obtained an overall classification accuracy of 0.8 or higher, the results of which are shown in Fig. 8. To quantify feature importance, model-specific metrics, the coefficients of the features in the RLR model, the mean decrease impurity (Gini importance) of the RF model, the gain measures of the XGB model, and the absolute variable weights of the SVM model, were used. nPeaks was consistently identified as the one of the top two influential predictors in all six models, followed by another measure from the CTMT-B.

## IV. Discussion

This study developed a combined cognitive and sensorimotor evaluation system and conducted preliminary tests to quantify the performance differences among two age groups, using three proposed combined cognitive and sensorimotor tests. Our results confirmed that the older subjects exhibited both significantly decreased cognitive and sensorimotor performance. We also developed and compared classification models attempting to differentiate two age groups using eye movement and/or sensorimotor measures. The feature importance was analyzed to determine the most influential ones after the best-performing models were confirmed.

### A. Results of Combined Cognitive and Sensorimotor Evaluation

Although saccadic eye movements are believed to provide promising biometrics to assess cognitive function [25], [40], the reported comparison of correct eye movement latencies during AST between MCI/ADRD patients and controls varies; Wilcockson et al. [13] observed significantly enlarged latency of the patients, whereas Opwonya et al. [41] reported no differences were found between patients and controls. EyeAST did not differ significantly between older and young participants; the moderate effect size (Cohen’s d = 0.68) suggests a potential age-related difference that may not have reached significance due to limited sensitivity after stringent multiple-comparison correction. However, during CASMT, the older subjects exhibited significantly longer EyeCASMT than the young subjects; furthermore, older subjects showed significantly increased correct eye movement latency during CASMT compared with AST, whereas no significant changes in young subjects were detected. The 72.1ms increase in EyeCASMT latency in older adults may reflect a dual-task cost, indicating additional processing time required to manage concurrent cognitive and motor demands. This finding supports the rationale that combined tasks place greater demands on shared neural resources [42]. The increased latency may be related to age-associated declines in inhibitory control, attentional allocation, set-shifting, and sensorimotor integration, which depend on prefrontal, frontoparietal, cerebellar, and parietal networks [43], [44], [45]. The increase in WristCASMT latency (*p* = 0.014), although not significant after multiple-comparison correction, suggests that older adults may also experience motor-level slowing during dual-task performance. The parallel slowing of eye and wrist responses supports the interpretation that aging affects both cognitive and motor components of combined response behaviors, supporting that the proposed CASMT can enhance the detection of cognitive changes.

Our findings were consistent with previous studies where slower reaction times in tasks requiring visual-motor integration of older adults were reported [46], [47], [48]. These declines are further compounded in multitasking scenarios, where age-related reductions in working memory and attentional control impede the ability to manage concurrent cognitive and motor demands [49], [50]. This can be explained by the fact that eye and limb motions are highly coupled during visuomotor activities [47], [48], [51], [52], and dual tasking (combined cognitive and motor tasks) leads to a significantly increased cognitive challenge for older adults and patients with cognitive impairment [50]. Studies on the extended Stroop effect, in which motor responses or dual-task conditions are incorporated, also report that older adults experience greater cognitive interference and exhibit slower performance compared to young adults, underscoring the challenges of integrating cognitive and motor processes in aging populations [53], [54], [55]. Older subjects’ worse cognitive and motor performance than the young subjects during the CTMT-B could also be attributed to the above reasons.

The proposed tests also involved the sensory evaluation of the ability to detect movement because the proprioception function is confounded with cognitive function due to the shared neural pathway and cortical area [56]. The WristCASST reflects subjects’ proprioceptive function and the EyeCASST embodies cognitive function because it is related to attention and executive performance. Significantly increased WristCASST and EyeCASST of the older subjects were found which endorses the efficiency of CASST in the detection of cognitive differences.

### B. Metric Correlation Analysis

The correlation coefficients between each pair of measures were analyzed in this study. Results showed a significant positive correlation between EyeCASMT and WristCASMT in young participants, but a negligible correlation in older participants, suggesting age-related decoupling of eye-hand coordination under high cognitive demand. This pattern is consistent with evidence that aging reduces the efficiency of parallel visuomotor processing, particularly during rapid perception-action decisions [57], [58]. Under the demanding CASMT condition, older adults may shift from a coupled, parallel visuomotor strategy to a more sequential response mode, prioritizing visual-cognitive evaluation before initiating the motor response [59]. This shift may reflect age-related declines in executive control and sensorimotor integration [47]. In contrast, older and young participants showed similar EyeCASST and WristCASST correlations, likely because CASST uses a gradual proprioceptive stimulus that allows more predictable, self-timed responses. Under this lower cognitive load, eye and wrist responses may be driven by shared sensory cues and convergent visuomotor pathways involving the posterior parietal cortex and cerebellum [23], [24]. These findings suggest that age-related eye-hand decoupling becomes more apparent when rapid and unpredictable stimuli increase executive-control demands [57], [58], [59], [60].

Significant negative correlations between MoCA scores and selected CASMT and CTMT-B measures support the cognitive relevance of the proposed metrics. Consistent with the group-level finding that young participants had higher MoCA scores, these associations suggest that lower cognitive screening performance is linked to reduced efficiency in combined-task performance. These findings support the sensitivity of the proposed framework to age-related cognitive variability and motivate future validation in MCI/ADRD cohorts.

### C. Insights From the Classification Models

Four classic classifiers were employed to build models to map the relationship between the proposed outcome measures and age groups. Results suggested that the SM set outperformed the EM set, which supports the idea that human sensorimotor changes are promising potential biomarkers for cognitive declines [8] and confirms that the combined multiple assessment modalities can produce more comprehensive insights in enhancing the diagnosis and classification of mental diseases [15], [16], [17]. Among the five SM measures, features extracted from CTMT-B, including nPeaks, tDwell, and tReach, are more critical (Fig. 8), which can be attributed to CTMT-B inducing higher cognitive and motor workload than the others and increased workload enlarging the performance differences between age groups [50], [60]. Interestingly, the models with all measures did not achieve better performance; this may be caused by the overfitting problems when irrelevant features are involved in the models [61]. The feature importance analysis of the models with all measures identified two EM measures, including nReach and EyeCASMT, as one of the top three influential predictors (Fig. 8), which reiterates the results that measures from CTMT-B are more influential and the proposed CASMT can improve the detection of cognitive changes. The tDwell and tReach were also found to be top influential factors in all models in Fig. 8, which is reasonable, because, although tDwell and tReach belong to SM, it can also reflect subjects’ working memory and attention, as when placing the hand-cursor at the target and moving it between targets, the subjects are searching for the next correct object and planning the next motion. In summary, outcome measures from CTMT-B were confirmed as distinguished influential features.

The stronger discriminative performance of SM metrics, particularly nPeaks, may reflect the sensitivity of motor planning and execution to age-related changes. Unlike cognitive latency, which primarily reflects inhibitory control and stimulus evaluation, nPeaks captures detailed motor-output features such as sub-movement decomposition, trajectory corrections, and neuromuscular stability. These processes rely on cerebellar, basal ganglia, parietal, and corticospinal networks that decline with age [47]. Thus, sensorimotor metrics may serve as more sensitive biomarkers of aging by capturing deficits across motor planning, executive control, and sensorimotor integration. While the current results suggest that models using SM measures performed the best, it is important to note that Fig. 8e&f indicates eye movement measures such as nReach and EyeCASMT also played significant roles, suggesting that EM measures may also contain valuable information. Given the limited sample size in this study, an exhaustive search for the optimal measure combination was not feasible. However, we anticipate that future studies with larger participant cohorts may result in a best set of predictors including a combination of both cognitive and sensorimotor measures.

### D. Comparison With Other Dual-Tasks and Future Work

Researchers have also investigated the application of other dual-tasks in quantifying the status of cognition of older adults, such as gait-based [20], [62], [63] and upper-extremity function-based [19], [50], [64] approaches and reported high sensitivity and specificity in identifying cognitive status. These studies demonstrate that combined cognitive and motor tasks can reveal declines not captured by single-task tests alone, consistent with the present finding that integrated metrics differentiate young and older adults better than the cognitive test alone. However, those methods are physically demanding and may lead to high fall risks for older adults, hindering their applicability in clinical settings; the cognitive tasks they used are partial of the neuropsychological test batteries which can provide limited information of participants’ executive and sensorimotor functions involved in daily activities. Our proposed tasks utilize simple wrist motions and non-strenuous arm movements, making them safer and more accessible. Integrating an arm-hand exoskeleton with eye tracking enables synchronized, high-precision measurement of eye and armhand movements, allowing detection of age-related changes in visuomotor integration, proprioceptive reactivity, and eye-hand coordination that prior dual-task studies could not directly capture. Our findings extend prior work by showing that aging affects not only motor output or cognitive timing, but also interactions among sensory, motor, and executive processes. Although the current system is bulky and requires technical support, it is intended as a proof-of-concept platform rather than a final deployable screening device. Its near-term niche may be as a quantitative, non-invasive functional assessment tool for rehabilitation hospitals and research settings where detailed characterization of cognitive-sensorimotor function is needed. In these settings, the system could complement brief cognitive screening by providing objective measures of dual-task coordination, visuomotor integration, proprioceptive response, and motor execution quality. Future translation will require replacing the laboratory exoskeleton with a lightweight wrist/hand module, using contactless eye tracking, and automating calibration, task administration, and data analysis to reduce cost and technical burden. Thus, while the current system is unlikely to be suitable for broad primary-care screening, it may provide value for specialized screening, longitudinal monitoring, and intervention planning where detailed functional biomarkers are needed.

Moreover, combined cognitive and physical training has been reported to outperform cognitive or physical intervention alone in promoting the cognitive function of older adults [65], [66], [67]. Our proposed system and tests can also be applied to conduct combined cognitive and sensorimotor training for older adults with cognitive impairment (see details in the following paragraph). Considering the light physical demands of our proposed tests, we anticipate that our approach can be more appealing than protocols involving aerobic and strength training, e.g., Nishiguchi et al. [68], guaranteeing the adherence rate of the training [69]. However, more studies are warranted to investigate the performance of the proposed method in slowing down cognitive declines.

Although the present study focused on healthy young and older adults, the combined cognitive and sensorimotor tests and derived metrics are designed to support a phased translational pathway toward assessment and rehabilitation training in individuals with MCI/ADRD. In the first phase, feasibility and cross-sectional sensitivity would be evaluated by applying the developed procedures to MCI/ADRD cohorts and age-matched controls, with particular attention to Dual-Task Cost and sensorimotor metrics such as nPeaks, tDwell, and cursor movement smoothness. We hypothesize that, compared to cognitively healthy older adults, individuals with MCI/ADRD would exhibit larger Dual-Task Costs, reflecting disproportionate slowing under combined cognitive-motor load, and higher nPeaks and longer tDwell, indicating more fragmented motor execution and prolonged dwell times on intermediate locations during set-shifting. We have already initiated this phase study by involving MCI/ADRD patients. In the second phase, longitudinal studies could examine whether baseline Dual-Task Cost and other developed metrics predict subsequent cognitive decline or functional outcomes, thereby testing their value as prognostic biomarkers. We will also include validation against established dementia biomarker-based reference standards, such as structural and functional neuroimaging measures and CSF or plasma biomarkers. Benchmarking the predictive performance of the proposed cognitive-sensorimotor metrics against these established markers would help determine whether the system provides complementary information for identifying MCI/ADRD risk, monitoring disease progression, and supporting clinical decision-making. This validation would be essential for solidifying the clinical utility of the proposed system as a non-invasive, accessible screening and monitoring tool. The third phase would focus on structured training protocols that target the deficits identified by these metrics. For example, individuals with elevated nPeaks could engage in gamified graded sensorimotor training that emphasizes smoother continuous hand (cursor) movement trajectories with gradually increasing complexity, supported by real-time feedback on movement smoothness. Changes in Dual-Task Cost and other metrics would be used both as outcome measures and as indicators for personalizing the level and focus of training.

### E. Limitations

Several limitations should be acknowledged. First, the small sample size limited statistical power, despite preliminary evidence supporting the effectiveness of the proposed approach for detecting age-related cognitive differences. The reduced CTMT-B sample due to missing data further increased uncertainty in interpreting CTMT-B-related metrics. In addition, participants were relatively homogeneous in educational background, with many recruited from the University System of Maryland, which may limit generalizability. Future studies should recruit larger and more demographically diverse samples to validate these findings, support more advanced machine-learning approaches, and enable the use of an independent test set for more generalizable performance evaluation. Second, this preliminary analysis focused on a targeted set of established measures to reduce the risk of Type I error given the limited number of participants and trials. Although inhibitory error rate during AST has been shown to reflect cognitive status [12], [13], [14], the limited repetitions in this study may reduce the reliability of error-rate estimates. Other eye-movement measures, such as pupil diameter, blink rate, and gaze trajectory, may also provide additional insight into cognitive load and aging effects [70], [71]. Future studies with larger samples and more trials will examine these measures, along with wrist-movement error rates, to identify additional biomarkers. Finally, the clinical relevance of the findings remains to be established because the current study included only healthy young and older adults. Future work will recruit more healthy older adults and individuals with MCI/ADRD to determine whether the proposed measures can generalize to populations with clinically relevant cognitive decline.

## V. Conclusion

This study developed a combined cognitive and sensorimotor evaluation system and three combined cognitive and sensorimotor tests. The results showed that the combined cognitive and sensorimotor tests improve detection of cognitive and sensorimotor changes. Four machine-learning models were used to classify participants’ age groups based on the extracted multimodal features. The results showed that CTMT-B-derived features were important contributors to model performance, supporting the value of the proposed combined multimodal tests for detecting age-related cognitive-sensorimotor differences. However, the current findings should be interpreted as evidence of sensitivity to age-related cognitive-sensorimotor differences rather than clinical diagnostic validation. Future studies involving age-matched participants with varying cognitive status are needed to determine the system’s utility for detecting MCI/ADRD, monitoring cognitive decline, and conducting early intervention training using the same system.

## Supplementary Material

supp1-3711558

This article has supplementary downloadable material available at https://doi.org/10.1109/TNSRE.2026.3711558, provided by the authors.

## Figures and Tables

**TABLE I:** Pearson’s Correlation Coefficients Between the Proposed Measures and MoCA Score

	EyeAST	EyeCASMT	WristCASMT	EyeCASST	WristCASST

*r*	−0.04	−0.47	−0.26	−0.30	−0.24
*p-value*	0.834	0.006	0.150	0.101	0.186

	tReach	tDwell	nReach	nDwell	nPeaks

*r*	−0.29	−0.45	−0.33	−0.46	−0.43
*p-value*	0.110	0.012	0.078	0.011	0.017

**TABLE II:** Performance of Four Classification Models Using Three Outcome Measure Sets

Measures	Ridge Logistic Regression		Random Forest		Support Vector Machine		Extreme Gradient Boosting	

	Accuracy	F1	Accuracy	F1	Accuracy	F1	Accuracy	F1

EM	0.77	0.75	0.77	0.75	0.77	0.74	0.67	0.66
SM	0.80	0.77	0.87	0.83	0.83	0.83	0.87	0.87
All	0.80	0.77	0.73	0.72	0.77	0.74	0.80	0.77

## References

[1] Better MA, “Alzheimer’s disease facts and figures,” Alzheimers Dement, vol. 19, no. 4, pp. 1598–1695, Apr. 2023. 36918389 10.1002/alz.13016

[2] Livingston G , “Dementia prevention, intervention, and care,” Lancet, vol. 390, no. 10113, pp. 2673–2734, 2017. 28735855 10.1016/S0140-6736(17)31363-6

[3] Jack CR , “NIA-AA research framework: Toward a biological definition of Alzheimer’s disease,” Alzheimer’s Dementia, vol. 14, no. 4, pp. 535–562, Apr. 2018. 10.1016/j.jalz.2018.02.018 PMC5958625 29653606

[4] Bradfield NI, “Mild cognitive impairment: Diagnosis and subtypes,” Clin. EEG Neurosci., vol. 54, no. 1, pp. 4–11, Jan. 2023. 34549629 10.1177/15500594211042708

[5] McKhann GM , “The diagnosis of dementia due to Alzheimer’s disease: Recommendations from the National Institute on Aging-Alzheimer’s Association workgroups on diagnostic guidelines for Alzheimer’s disease,” Alzheimers Dement, vol. 7, no. 3, pp. 263–269, May 2011. 21514250 10.1016/j.jalz.2011.03.005 PMC3312024

[6] Nichols E , “Global, regional, and national burden of Alzheimer’s disease and other dementias, 1990–2016: A systematic analysis for the global burden of disease study 2016,” Lancet Neurol., vol. 18, no. 1, pp. 88–106, 2019. 30497964 10.1016/S1474-4422(18)30403-4 PMC6291454

[7] Shumway-Cook A and Woollacott MH, Motor Control: Translating Research Into Clinical Practice, 3rd ed. Philadelphia, PA, USA: Lippincott Williams & Wilkins, 2007.

[8] Albers MW , “At the interface of sensory and motor dysfunctions and Alzheimer’s disease,” Alzheimer’s Dementia, vol. 11, no. 1, pp. 70–98, 2015. 10.1016/j.jalz.2014.04.514 PMC4287457 25022540

[9] Wilson RS, Schneider JA, Bienias JL, Evans DA, and Bennett DA, “Parkinsonianlike signs and risk of incident Alzheimer disease in older persons,” Arch. Neurol., vol. 60, no. 4, p. 539, Apr. 2003. 12707067 10.1001/archneur.60.4.539

[10] Devanand DP , “Combining early markers strongly predicts conversion from mild cognitive impairment to Alzheimer’s disease,” Biol. Psychiatry, vol. 64, no. 10, pp. 871–879, Nov. 2008. 18723162 10.1016/j.biopsych.2008.06.020 PMC2613777

[11] Gates GA, Anderson ML, Feeney MP, McCurry SM, and Larson EB, “Central auditory dysfunction in older persons with memory impairment or Alzheimer dementia,” Arch. Otolaryngology–Head Neck Surg., vol. 134, no. 7, p. 771, Jul. 2008. 10.1001/archotol.134.7.771 PMC2871110 18645130

[12] Holden JG , “Prodromal Alzheimer’s disease demonstrates increased errors at a simple and automated anti-saccade task,” J. Alzheimer’s Disease, vol. 65, no. 4, pp. 1209–1223, Sep. 2018. 30149445 10.3233/JAD-180082

[13] Wilcockson TD , “Abnormalities of saccadic eye movements in dementia due to Alzheimer’s disease and mild cognitive impairment,” Aging (Albany NY), vol. 11, no. 15, p. 5389, 2019. 31375642 10.18632/aging.102118 PMC6710064

[14] Opwonya J, Ku B, Lee KH, Kim JI, and Kim JU, “Eye movement changes as an indicator of mild cognitive impairment,” Frontiers Neurosci., vol. 17, Jun. 2023, Art. no. 1171417. 10.3389/fnins.2023.1171417 PMC10307957 37397453

[15] Brooks LG and Loewenstein DA, “Assessing the progression of mild cognitive impairment to Alzheimer’s disease: Current trends and future directions,” Alzheimer’s Res. Therapy, vol. 2, no. 5, p. 28, Oct. 2010. 10.1186/alzrt52 PMC2983437 20920147

[16] Chipi E, Salvadori N, Farotti L, and Parnetti L, “Biomarker-based signature of Alzheimer’s disease in pre-MCI individuals,” Brain Sci., vol. 9, no. 9, p. 213, Aug. 2019. 31450744 10.3390/brainsci9090213 PMC6769621

[17] Martínez-Torteya A, Treviño V, and Tamez-Peña JG, “Improved diagnostic multimodal biomarkers for Alzheimer’s disease and mild cognitive impairment,” BioMed Res. Int., vol. 2015, May 2015, Art. no. 961314. 26106620 10.1155/2015/961314 PMC4464003

[18] Montero-Odasso M, Muir SW, and Speechley M, “Dual-task complexity affects gait in people with mild cognitive impairment: The interplay between gait variability, dual tasking, and risk of falls,” Arch. Phys. Med. Rehabil., vol. 93, no. 2, pp. 293–299, Feb. 2012. 22289240 10.1016/j.apmr.2011.08.026

[19] Toosizadeh N , “Upper-extremity dual-task function: An innovative method to assess cognitive impairment in older adults,” Frontiers Aging Neurosci., vol. 8, p. 167, Jul. 2016. 10.3389/fnagi.2016.00167 PMC4935727 27458374

[20] Lamoth CJ, van Deudekom FJ, van Campen JP, Appels BA, de Vries OJ, and Pijnappels M, “Gait stability and variability measures show effects of impaired cognition and dual tasking in frail people,” J. Neuroeng. Rehabil., vol. 8, no. 1, p. 2, Jan. 2011. 21241487 10.1186/1743-0003-8-2 PMC3034676

[21] Plummer-D’Amato P, Altmann LJP, Saracino D, Fox E, Behrman AL, and Marsiske M, “Interactions between cognitive tasks and gait after stroke: A dual task study,” Gait Posture, vol. 27, no. 4, pp. 683–688, May 2008. 17945497 10.1016/j.gaitpost.2007.09.001 PMC2913384

[22] Ren Y, Kang SH, Park H-S, Wu Y-N, and Zhang L-Q, “Developing a multi-joint upper limb exoskeleton robot for diagnosis, therapy, and outcome evaluation in neurorehabilitation,” IEEE Trans. Neural Syst. Rehabil. Eng., vol. 21, no. 3, pp. 490–499, May 2013. 23096119 10.1109/TNSRE.2012.2225073

[23] Andersen RA and Buneo CA, “Intentional maps in posterior parietal cortex,” Annu. Rev. Neurosci., vol. 25, no. 1, pp. 189–220, Mar. 2002. 12052908 10.1146/annurev.neuro.25.112701.142922

[24] Ramnani N, “The primate cortico-cerebellar system: Anatomy and function,” Nature Rev. Neurosci., vol. 7, no. 7, pp. 511–522, Jul. 2006. 16791141 10.1038/nrn1953

[25] Kusne Y, Wolf AB, Townley K, Conway M, and Peyman GA, “Visual system manifestations of Alzheimer’s disease,” Acta Ophthalmologica, vol. 95, no. 8, pp. e668–e676, Dec. 2017. 27864881 10.1111/aos.13319

[26] Yin W, Oppizzi G, Huang MZ, Koh K, Hur S-H, and Zhang L-Q, “Combined cognitive and sensorimotor assessment enhances detection of cognitive declines,” in Proc. Biomed. Eng. Soc. Annu. Meeting, Baltimore, MD, USA, 2024.

[27] Zhang L-Q , “Intelligent stretching of ankle joints with contracture/spasticity,” IEEE Trans. Neural Syst. Rehabil. Eng., vol. 10, no. 3, pp. 149–157, Sep. 2002. 12503779 10.1109/TNSRE.2002.802857

[28] Kang SH, Jin M, and Chang PH, “A solution to the accuracy/robustness dilemma in impedance control,” IEEE/ASME Trans. Mechatronics, vol. 14, no. 3, pp. 282–294, Jun. 2009.

[29] Nasreddine ZS , “The Montreal cognitive assessment, MoCA: A brief screening tool for mild cognitive impairment,” J. Amer. Geriatrics Soc., vol. 53, no. 4, pp. 695–699, Apr. 2005. 10.1111/j.1532-5415.2005.53221.x 15817019

[30] Xu D, Kang SH, Lee SJ, Oppizzi G, and Zhang L-Q, “Multi-joint assessment of proprioception impairments poststroke,” Arch. Phys. Med. Rehabil., vol. 105, no. 3, pp. 480–486, Mar. 2024. 37714505 10.1016/j.apmr.2023.08.029 PMC10922066

[31] Lee SJ, Ren Y, Chang AH, Geiger F, and Zhang L-Q, “Effects of pivoting neuromuscular training on pivoting control and proprioception,” Med. Sci. Sports Exercise, vol. 46, no. 7, pp. 1400–1409, 2014. 10.1249/MSS.0000000000000249 PMC4140528 24389517

[32] Lee SJ, Ren Y, Kang SH, Geiger F, and Zhang L-Q, “Pivoting neuromuscular control and proprioception in females and males,” Eur. J. Appl. Physiol., vol. 115, no. 4, pp. 775–784, Apr. 2015. 25431130 10.1007/s00421-014-3062-z

[33] Chang AH, Lee SJ, Zhao H, Ren Y, and Zhang L-Q, “Impaired varus–valgus proprioception and neuromuscular stabilization in medial knee osteoarthritis,” J. Biomech., vol. 47, no. 2, pp. 360–366, Jan. 2014. 24321442 10.1016/j.jbiomech.2013.11.024 PMC3929588

[34] Lee Y, Xu D, Ren Y, Chen K, Gaebler-Spira D, and Zhang L-Q, “Changes in ankle joint proprioception resulting from robotic training of ankle mobility rehabilitation in children with cerebral palsy,” presented at the Annu. Meeting Soc. Neurosci., Chicago, IL, USA, Oct. 2015.

[35] Kim M and Collins SH, “Once-per-step control of ankle-foot prosthesis push-off work reduces effort associated with balance during walking,” J. Neuroeng. Rehabil., vol. 12, no. 1, p. 43, May 2015. 25928176 10.1186/s12984-015-0027-3 PMC4429504

[36] Yin W, Reddy C, Zhou Y, and Zhang X, “A novel application of flexible inertial sensors for ambulatory measurement of gait kinematics,” IEEE Trans. Human-Mach. Syst., vol. 51, no. 4, pp. 346–354, Aug. 2021.

[37] Mardia KV, “Applications of some measures of multivariate skewness and kurtosis in testing normality and robustness studies,” Sankhyā: Indian J. Statist., B, vol. 36, no. 2, pp. 115–128, 1974.

[38] Royston P, “Approximating the shapiro-wilk W-test for non-normality,” Statist. Comput., vol. 2, no. 3, pp. 117–119, Sep. 1992.

[39] Kuhn M, “Building predictive models in r using the caret package,” J. Stat. Softw., vol. 28, no. 5, pp. 1–26, 2008.27774042

[40] Anderson TJ and MacAskill MR, “Eye movements in patients with neurodegenerative disorders,” Nature Rev. Neurol., vol. 9, no. 2, pp. 74–85, Feb. 2013. 23338283 10.1038/nrneurol.2012.273

[41] Opwonya J, “Inhibitory control of saccadic eye movements and cognitive impairment in mild cognitive impairment,” Alzheimer’s Dementia, vol. 18, no. S6, Dec. 2022, Art. no. 871432. 10.3389/fnagi.2022.871432 PMC9038187 35478701

[42] Marois R and Ivanoff J, “Capacity limits of information processing in the brain,” Trends Cognit. Sci., vol. 9, no. 6, pp. 296–305, Jun. 2005. 15925809 10.1016/j.tics.2005.04.010

[43] Buckner RL, “Memory and executive function in aging and AD: Multiple factors that cause decline and reserve factors that compensate,” Neuron, vol. 44, no. 1, pp. 195–208, Sep. 2004. 15450170 10.1016/j.neuron.2004.09.006

[44] Mayr U, “Age differences in the selection of mental sets: The role of inhibition, stimulus ambiguity, and response-set overlap,” Psychol. Aging, vol. 16, no. 1, pp. 96–109, Mar. 2001. 11302371 10.1037/0882-7974.16.1.96

[45] Bernard JA and Seidler RD, “Moving forward: Age effects on the cerebellum underlie cognitive and motor declines,” Neurosci. Biobehav. Rev., vol. 42, pp. 193–207, May 2014. 24594194 10.1016/j.neubiorev.2014.02.011 PMC4024443

[46] Van Halewyck F, Lavrysen A, Levin O, Boisgontier MP, Elliott D, and Helsen WF, “Both age and physical activity level impact on eye-hand coordination,” Hum. Movement Sci., vol. 36, pp. 80–96, Aug. 2014. 10.1016/j.humov.2014.05.005 24964357

[47] Seidler RD , “Motor control and aging: Links to age-related brain structural, functional, and biochemical effects,” Neurosci. Biobehav. Rev., vol. 34, no. 5, pp. 721–733, Apr. 2010. 19850077 10.1016/j.neubiorev.2009.10.005 PMC2838968

[48] Krampe RT, “Aging, expertise and fine motor movement,” Neurosci. Biobehav. Rev., vol. 26, no. 7, pp. 769–776, Nov. 2002. 12470688 10.1016/s0149-7634(02)00064-7

[49] Verhaeghen P, Steitz DW, Sliwinski MJ, and Cerella J, “Aging and dual-task performance: A meta-analysis,” Psychol. Aging, vol. 18, no. 3, pp. 443–460, Sep. 2003. 14518807 10.1037/0882-7974.18.3.443

[50] Ehsani H, Mohler MJ, O’Connor K, Zamrini E, Tirambulo C, and Toosizadeh N, “The association between cognition and dual-tasking among older adults: The effect of motor function type and cognition task difficulty,” Clin. Interventions Aging, vol. 14, pp. 659–669, Apr. 2019. 10.2147/CIA.S198697 PMC6459153 31040655

[51] Neggers SFW and Bekkering H, “Ocular gaze is anchored to the target of an ongoing pointing movement,” J. Neurophysiol., vol. 83, no. 2, pp. 639–651, Feb. 2000. 10669480 10.1152/jn.2000.83.2.639

[52] Rand MK and Stelmach GE, “Adaptation of gaze anchoring through practice in young and older adults,” Neurosci. Lett., vol. 492, no. 1, pp. 47–51, Mar. 2011. 21276832 10.1016/j.neulet.2011.01.051 PMC3046210

[53] Perrochon A, Kemoun G, Watelain E, and Berthoz A, “Walking stroop carpet: An innovative dual-task concept for detecting cognitive impairment,” Clin. Interventions Aging, vol. 8, pp. 317–328, Mar. 2013. 10.2147/CIA.S38667 PMC3610448 23682211

[54] Ward N, Hussey E, Alzahabi R, Gaspar JG, and Kramer AF, “Age-related effects on a novel dual-task stroop paradigm,” PLoS ONE, vol. 16, no. 3, Mar. 2021, Art. no. e0247923. 33651855 10.1371/journal.pone.0247923 PMC7924780

[55] Peskar M, Omejc N, Šömen MM, Miladinović A, Gramann K, and Marusic U, “Stroop in motion: Neurodynamic modulation underlying interference control while sitting, standing, and walking,” Biol. Psychol., vol. 178, Mar. 2023, Art. no. 108543. 36931590 10.1016/j.biopsycho.2023.108543

[56] Piitulainen H, Seipäjärvi S, Avela J, Parviainen T, and Walker S, “Cortical proprioceptive processing is altered by aging,” Frontiers Aging Neurosci., vol. 10, p. 147, Jun. 2018. 10.3389/fnagi.2018.00147 PMC6010536 29962945

[57] Gómez-Granados A, Barany DA, Schrayer M, Kurtzer IL, Bonnet CT, and Singh T, “Age-related deficits in rapid visuomotor decision-making,” J. Neurophysiol., vol. 126, no. 5, pp. 1592–1603, Nov. 2021. 34614375 10.1152/jn.00073.2021

[58] Guan J and Wade MG, “The effect of aging on adaptive eye-hand coordination,” J. Gerontol. B, Psychol. Sci. Social Sci., vol. 55, no. 3, pp. P151–P162, May 2000. 10.1093/geronb/55.3.p151 11833977

[59] Kwon M and Christou EA, “Visual information processing in older adults: Reaction time and motor unit pool modulation,” J. Neurophysiol., vol. 120, no. 5, pp. 2630–2639, Nov. 2018. 30207861 10.1152/jn.00161.2018 PMC6295548

[60] Qu X, “Age-related cognitive task effects on gait characteristics: Do different working memory components make a difference?” J. Neuroeng. Rehabil., vol. 11, no. 1, p. 149, 2014. 25348927 10.1186/1743-0003-11-149 PMC4221663

[61] Yin W, Chen Y, Reddy C, and Zhang X, “Towards real-time minimum-input prediction of lumbar moment based on flexible sensors and machine learning,” in Proc. Hum. Factors Ergonom. Soc. 66th Int. Annu. Meeting, Atlanta, GA, USA, 2022, pp. 656–660.

[62] Doi T , “Cognitive function and gait speed under normal and dual-task walking among older adults with mild cognitive impairment,” BMC Neurol., vol. 14, no. 1, p. 67, Apr. 2014. 24694100 10.1186/1471-2377-14-67 PMC3994221

[63] IJmker T and Lamoth CJC, “Gait and cognition: The relationship between gait stability and variability with executive function in persons with and without dementia,” Gait Posture, vol. 35, no. 1, pp. 126–130, Jan. 2012. 21964053 10.1016/j.gaitpost.2011.08.022

[64] Toosizadeh N, Mohler J, and Najafi B, “Assessing upper extremity motion: An innovative method to identify frailty,” J. Amer. Geriatrics Soc., vol. 63, no. 6, pp. 1181–1186, Jun. 2015. 10.1111/jgs.13451 PMC6653678 26096391

[65] H. M. Gavelin , “Combined physical and cognitive training for older adults with and without cognitive impairment: A systematic review and network meta-analysis of randomized controlled trials,” Ageing Res. Rev., vol. 66, Mar. 2021, Art. no. 101232. 33249177 10.1016/j.arr.2020.101232

[66] Zhu X, Yin S, Lang M, He R, and Li J, “The more the better? A meta-analysis on effects of combined cognitive and physical intervention on cognition in healthy older adults,” Ageing Res. Rev., vol. 31, pp. 67–79, Nov. 2016. 27423932 10.1016/j.arr.2016.07.003

[67] Chan P-T , “Effect of interactive cognitive-motor training on eye-hand coordination and cognitive function in older adults,” BMC Geriatrics, vol. 19, no. 1, p. 27, Jan. s2019. 30691404 10.1186/s12877-019-1029-y PMC6350349

[68] Nishiguchi S , “A 12-week physical and cognitive exercise program can improve cognitive function and neural efficiency in community-dwelling older adults: A randomized controlled trial,” J. Amer. Geriatrics Soc., vol. 63, no. 7, pp. 1355–1363, Jul. 2015. 10.1111/jgs.13481 26114906

[69] Farrance C, Tsofliou F, and Clark C, “Adherence to community based group exercise interventions for older people: A mixed-methods systematic review,” Preventive Med., vol. 87, pp. 155–166, Jun. 2016. 10.1016/j.ypmed.2016.02.037 26921655

[70] Zahabi M, Shahini F, Yin W, and Zhang X, “Physical and cognitive demands associated with police in-vehicle technology use: An on-road case study,” Ergonomics, vol. 65, no. 1, pp. 1–34, Jan. 2022. 34308789 10.1080/00140139.2021.1960429

[71] El Haj M, “Pupillometry as tool to assess cognitive and affective processing in aging,” Brain Disorders, vol. 14, Jun. 2024, Art. no. 100129.

