# Supplementary material for: Combining Cognitive and Sensorimotor Assessments to Facilitate Detection of Age-Related Cognitive Decline
Source: IEEE Trans Neural Syst Rehabil Eng. Author manuscript; Available in PMC 2026 Aug 11. (PMC13461145; doi:10.1109/TNSRE.2026.3711558)
Supplement: Supplementary file 1 [file NIHMS2196888-supplement-supp1-3711558.docx]

Supplementary Materials

### Outcome Measures

1. Detection of saccade onsets and wrist motion onsets

The saccadic data and the robot’s wrist joint torque profiles were used to derive outcome measures on subjects’ cognitive and sensorimotor performance. The default settings of EyeLink Data Viewer 4.2.1 (SR Research Ltd., Kanata, ON, Canada) were used to detect saccade onsets: a velocity above 30°/s and a deflection above 0.1°; through a pilot test, the thresholds for identifying the wrist motion onsets were set as a torque rising rate higher than 4Nm/s and a torque magnitude change exceeding 0.02Nm. Although joint stiffness may influence torque generation and rise rate [1], its impact was minimized because the wrist was held isometrically, with no joint displacement during torque generation [2]. Thus, the selected thresholds provided a consistent and physiologically meaningful marker for detecting voluntary torque onset across individuals with varying stiffness characteristics. An example showing the detection of onset of eye and wrist movement is shown in Fig. 5.

Fig. S1. An example of the estimation of eye and wrist movement latency. (a) the horizontal gaze location and (b) the wrist joint torque data (negative torque indicates wrist flexion). The example is from a CASMT trial.

2. Rationales of using two eccentricities for AST and CASMT

In both AST and CASMT, distractors were presented at two eccentricities, which may modestly affect saccadic latency. However, prior studies suggest that the eccentricities used here have relatively small effects on latency [3, 4]. Because this study focused on group differences in inhibitory control and dual-task responses rather than spatial effects, latency values were pooled across eccentricities. Although eccentricity may contribute to latency variability and will be modeled in future studies, pooling was unlikely to obscure age-related group differences given the study aims and limited eccentricity range.

3. Determination of cursor dwell and reach for CTMT-B

The hand-cursor (15 mm diameter) was considered on the target if any part of it fell in the interest area (60 mm diameter) around the target. An example of the identified cursor dwelling and reaching is shown in Fig. S2.

Fig. S2. An example of the determination of cursor dwelling and reaching during CTMT-B.

4. An example of the measurement of CTMT-B

Fig. S3. An example of the measurement of CTMT-B. The red curve represents the cursor movement trajectory; the heatmap shows the eye fixation duration and distribution. The picture was created by EyeLink Data Viewer 4.2.1, SR Research Ltd., Kanata, ON, Canada.

References:

[1]	A. Monte, “In vivo manipulation of muscle shape and tendinous stiffness affects the human ability to generate torque rapidly,” *Experimental Physiology,* vol. 106, no. 2, pp. 486-495, 2021.

[2]	L. L. Andersen, and P. Aagaard, “Influence of maximal muscle strength and intrinsic muscle contractile properties on contractile rate of force development,” *European Journal of Applied Physiology,* vol. 96, no. 1, pp. 46-52, 2006/01/01, 2006.

[3]	A. H. Bell, S. Everling, and D. P. Munoz, “Influence of stimulus eccentricity and direction on characteristics of pro- and antisaccades in non-human primates,” *J Neurophysiol,* vol. 84, no. 5, pp. 2595-604, Nov, 2000.

[4]	D. Mazumdar, N. S. K. Meethal, M. Panday, R. Asokan, G. Thepass, R. J. George, J. van der Steen, and J. J. M. Pel, “Effect of Age, Sex, Stimulus Intensity, and Eccentricity on Saccadic Reaction Time in Eye Movement Perimetry,” *Transl Vis Sci Technol,* vol. 8, no. 4, pp. 13, Jul, 2019.
